# Australian Pharmaceutical Benefits scheme data on utilisation of intravitreal therapy in retinal vein occlusion

**DOI:** 10.1111/ceo.14131

**Published:** 2022-08-05

**Authors:** Paul Mitchell, Prabhjot Juneja, Chao Wang, Rebecca Schnabel

**Affiliations:** ^1^ University of Sydney Sydney Australia; ^2^ Prospection Pty Ltd Sydney Australia; ^3^ Allergan, an AbbVie company Sydney Australia

Treatment of retinal vein occlusion (RVO) with monthly injections of anti‐vascular endothelial growth factor (VEGF) agents has been shown to improve visual outcomes,^1^ but real‐world data^2^, ^3^ suggest that some patients have suboptimal outcomes, despite receiving frequent injections. Given these considerations, we used the Australian Pharmaceutical Benefits Scheme (PBS) 10% data set to elucidate treatment patterns with VEGF inhibitors in RVO and to identify limitations of their use.

The PBS 10% data set contains records of all medicines dispensed to a 10% random sample of PBS‐eligible Australian residents. Data were extracted from 1 June 2015 to 31 December 2017 by Prospection Pty Ltd, the data custodian. The study and publication were approved by the Australian Government Department of Human Services External Request Evaluation Committee (Approval Number RMS 1424, 19 December 2020) and complied with relevant data protection and privacy regulations.

Included were patients who met Australian PBS reimbursement criteria for VEGF inhibitor treatment of branch RVO (BRVO), central RVO (CRVO), or both, initiated treatment between 1 June 2015 and 31 December 2016, and had follow‐up records for at least 1 year. Ranibizumab and aflibercept were the only anti‐VEGF therapies reimbursed through the PBS for RVO when the study was conducted. Prescriptions were identified using PBS item codes and relevant indication (Authority) codes. Anonymised data collected included medication and date prescribed, patient demographics, PBS item code and indication. The key outcome measure was the mean number of anti‐VEGF agents dispensed for RVO by treatment year over 2 years.

Overall, 681 patients (Table 1) had the required one‐year follow‐up data, and 332 of these patients met the criteria for 2‐year follow‐up data. Of the 681 patients, 569 (83.6%) received ranibizumab, 266 (39.1%) received aflibercept and 154 (22.6%) switched agents during treatment. An upward trend of aflibercept use was observed from 1 October 2015, the date of first PBS listing for CRVO.

**TABLE 1:** Patient demographics, locations of prescriptions dispensed at initiation of treatment for RVO, and type of RVO

Parameter	Full study cohort (*N* = 681)
Gender, *n* (%)	
Male	338 (49.6)
Female	343 (50.4)
Age at time dispensed	
Mean (*SD*)	72.5 (11.8)
Category, *n* (%)	
15–39	7 (1.0)
40–44	4 (0.6)
45–49	19 (2.8)
50–54	23 (3.4)
55–59	40 (5.9)
60–64	56 (8.2)
65–69	97 (14.2)
70–74	108 (15.9)
75–79	115 (16.9)
80–84	112 (16.4)
85–89	75 (11.0)
90+	25 (3.6)
State/territory of dispensing, *n* (%)	
New South Wales and Australian Capital Territory	311 (45.7)
Victoria and Northern Territory	138 (20.3)
Queensland	129 (18.9)
Western Australia	51 (7.5)
South Australia	30 (4.4)
Tasmania	22 (3.2)
Type of RVO, *n (%)*	
BRVO	421 (61.8)
CRVO	315 (46.3)
Both	55 (8.1)

*Note*: Relative proportions for patient demographics are shown. Age was calculated as the number of years from the year of birth to the year of initiating RVO treatment.

Abbreviation: RVO, retinal vein occlusion.

In treatment year 1, 448 (65.8%) patients received 6 or more anti‐VEGF injections (Figure 1A); 31.4% of patients received 9 or more injections, 34.4% received 6–8 injections, 26.4% received 3–5 injections and 7.8% received less than 3 injections. Distributions for BRVO and CRVO were generally consistent with those for the full population (Figure 1C,E).

**FIGURE 1:**
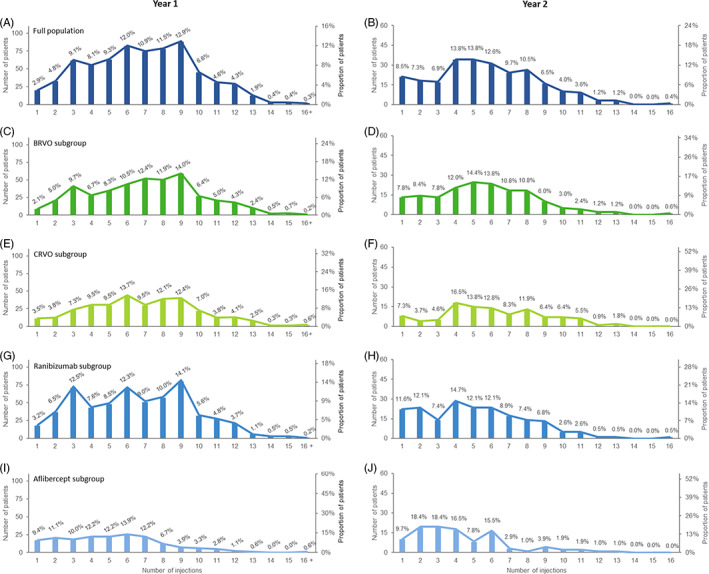
Numbers (bars) and proportions (lines) of injections received in (A) the first year of treatment in the full population (*N* = 681); (B) the second year of treatment in the subset of patients with 2‐year follow‐ up data (*N* = 247); (C and D) patients with BRVO in the first year of treatment (*n* = 421) and the second year of treatment in patients with 2‐year follow‐up (*n* = 167); (E and F) patients with CRVO in the first year of treatment (*n* = 315) and the second year of treatment in patients with 2‐year follow‐up (*n* = 109); (G and H) patients treated with ranibizumab in the first year of treatment (*n* = 568) and the second year of treatment in patients with 2‐year follow‐up (*n* = 190); (I and J) patients treated with aflibercept in the first year of treatment (*n* = 180) and the second year of treatment with 2‐year follow‐up (*n* = 103)

The pattern of VEGF inhibitor injections dispensed in year 1 in patients with 2‐year follow‐up (*n* = 332) was consistent with the full population (Figure 1A): 34.9% received 9 or more injections, 33.1% received 6–8 injections, 25.9% received 3–5 injections, and 6.0% received less than 3 injections in year 1. In year 2, 247 (74.4%) patients received VEGF inhibitors (Figure 1B). Approximately one half of patients who received treatment in year 2 received 6 or more injections, 17.0% received 9 or more injections, 32.8% received 6–8 injections, 34.4% received 3–5 injections, and 15.8% received less than 3 injections. Injection distribution in the BRVO and CRVO subgroups was similar (Figure 1D,F).

The mean ± *SD* number of injections/patient during year 1 was 6.9 ± 3.1 overall, and 6.6 ± 3.1 and 5.2 ± 2.9 in the ranibizumab (*n* = 568) and aflibercept (*n* = 180) subsets, respectively. The mean ± *SD* number of injections/patient during year 2 was 5.7 ± 2.9 overall, and 5.1 ± 2.9 and 4.3 ± 2.6 in the ranibizumab (*n* = 190) and aflibercept (*n* = 103) subsets, respectively. There was a general trend towards patients receiving more injections per year with increasing age, particularly for CRVO. The proportions of patients aged 60 years or older who received six or more injections were consistently high (67.0–68.7%) across indications.

Our results show that in real‐world practice, over two thirds of patients failed to achieve resolution of RVO in year 1, despite being prescribed six or more injections: 74.4% of patients underwent treatment beyond 1 year, with one‐half requiring six or more injections in year 2 of treatment. In general, the treatment burden in most patients with RVO was substantial. Comparable results have been reported in studies from other jurisdictions.^4^, ^5^ Failure to achieve resolution of RVO in year 1 of treatment may partly reflect the differences in injection frequencies in real‐world clinical practice compared with those in randomised clinical trials with defined monthly injection schedules.^1^

Although the current study includes data from a large database and a diverse patient population, limitations include size and sample of the data set, inability to generalise to other populations or health systems, Authority‐related drawbacks to identifying RVO diagnoses, underestimation of current aflibercept use, and lack of efficacy or safety data.

Despite these drawbacks, this study of a real‐world Australian population sample indicates that RVO may not resolve with anti‐VEGF despite relatively high treatment burdens. These data suggest a place for alternative treatments with different mechanisms of action and reduced frequency, such as sustained release corticosteroid implants. The present study did not capture the use of the dexamethasone intravitreal implant, which was PBS‐listed after the study period. Thus, current utilisation of this treatment option was not reflected.

## FUNDING INFORMATION

Allergan, an AbbVie company, funded this study and participated in the trial design, interpretation of the data, and the review and approval of the publication.

## CONFLICT OF INTEREST

Financial arrangements of the authors with companies whose products may be related to the present report are listed as declared by the authors: Paul Mitchell is a consultant for Allergan (an AbbVie company). Prabhjot Juneja was an employee of Prospection while the study was being conducted. Chao Wang is a full‐time employee of Prospection Pty Ltd. Prospection Pty Ltd was contracted by Allergan Pty Ltd (prior to its acquisition by AbbVie) for data collection and analysis. Rebecca Schnabel was an employee of Allergan plc during the time the study was conducted, and the manuscript was developed.
